# Editorial: Immunology of food-borne parasites: recent progress and future advances

**DOI:** 10.3389/fimmu.2025.1533086

**Published:** 2025-01-24

**Authors:** Saeed El-Ashram, Xi-Meng Sun, Gungor Cagdas Dincel, Xochitl Hernandez-Velasco, Yong-Sheng Ji

**Affiliations:** ^1^ Zoology Department, Faculty of Science, Kafrelsheikh University, Kafr El-Sheikh, Egypt; ^2^ College of Life Science and Engineering, Foshan University, Foshan, Guangdong, China; ^3^ Department of Medical Microbiology and Parasitology, School of Basic Medical Sciences, Capital Medical University, Beijing, China; ^4^ Department of Medical Pathology, Faculty of Medicine, Ankara Medipol University, Ankara, Türkiye; ^5^ Department of Avian Medicine and Zootechnics, College of Veterinary Medicine and Zootechnics, National Autonomous University of Mexico, Mexico City, Mexico; ^6^ Division of Life Sciences and Medicine, University of Science and Technology of China (USTC), Hefei, China

**Keywords:** food-borne parasites, immunity, vaccine, type I interferon, complement evasion

Parasites transmitted through food can be a severe concern to the health of the population around the world, producing millions of cases every year. The small aliens of this category embrace protozoa, helminths, and similar organisms. They can cause varied illnesses, ranging from minor gastrointestinal problems to deadly ailments. To find out treatment and preventive methods for this disease, it is important to research the immunological mechanisms of the body against them. These little invaders include protozoa, helminths, and other related organisms. These can cause a wide range of health issues, from certain stomach irritations to severe, life-threatening diseases. The inquiry into the immunological mechanisms of the body against these microorganisms is key in public health because it provides the grounds for new prevention methods and treatment of the illness. The editorial includes the latest findings in the food-borne parasite immune system, the most relevant results, current research trends, and the likely future prospects.

## Key findings and advancements

### *Toxoplasma gondii* and type I interferon

The *Toxoplasma gondii* is one of the most common food-borne parasites transmittable to most mammalian hosts; humans are no exception. Recent studies have illuminated the complex relationship between the *T. gondii* and the host immune system, particularly emphasizing Type I interferon (IFN-I) involvement. Even though IFN-I has been known for a long time as a major antiviral cytokine, the latest research results point to its essential role in limiting infection. The production and utilization of IFN-I during the *T. gondii* infection depend on several factors, such as cell type, parasite genotype, and mouse strain. The *T. gondii*, on the other hand, secretes proteins that change the IFN-I pathway and thus become part of its immune escapism techniques (Song et al.).

### Calreticulin and complement evasion

Calreticulin (CRT) is a highly versatile protein that can participate in several physiological processes, including calcium regulation and immune responses. The latest research has discovered that helminths, such as *Trichinella spiralis*, produce CRT, which controls the host’s complement system and thus enables immune evasion. The atomic structure of *T. spiralis* CRT has been elucidated, which shows how it binds to the C1q fragment and, therefore, prevents the formation of the traditional complement pathway. This structural finding is a reference for designing parasite-based peptides that can be used as potential vaccines or therapeutic agents for complement-related autoimmune disorders (Jia et al.).

### *Eimeria papillata* and oxidative stress

Food-borne parasites that cause coccidiosis are called *Eimeria* species. This study looked at how well crude plant extracts that can fight coccidiosis were tested for safety. Under a chromatographic analysis, it was detected that *Eimeria papillata* triggers the body’s oxidative stress, which causes it to produce more reactive oxygen species (ROS) and, in general, lipid peroxidation. Treating them with *K. lappacea* root extract reduced the oxidative damage and inflammation in the jejunum in acute Toxoplasma, as reported by Abdel-Gaber in 2024.

### Immune evasion strategies

The escape route from the host’s immune system has been developed by food-borne parasites. For example, *T. gondii* secretes effective proteins (including ROP18 and ROP16) that act on the IFN-I production pathway and downstream interferon-stimulated genes (ISGs) control in different ways. These proteins prevent the parasite’s destruction and allow it to form long-lasting infections. A better understanding of these evasion mechanisms is a critical prerequisite for the rational design of drugs and vaccines (1).

## Future directions

**Figure 1:**
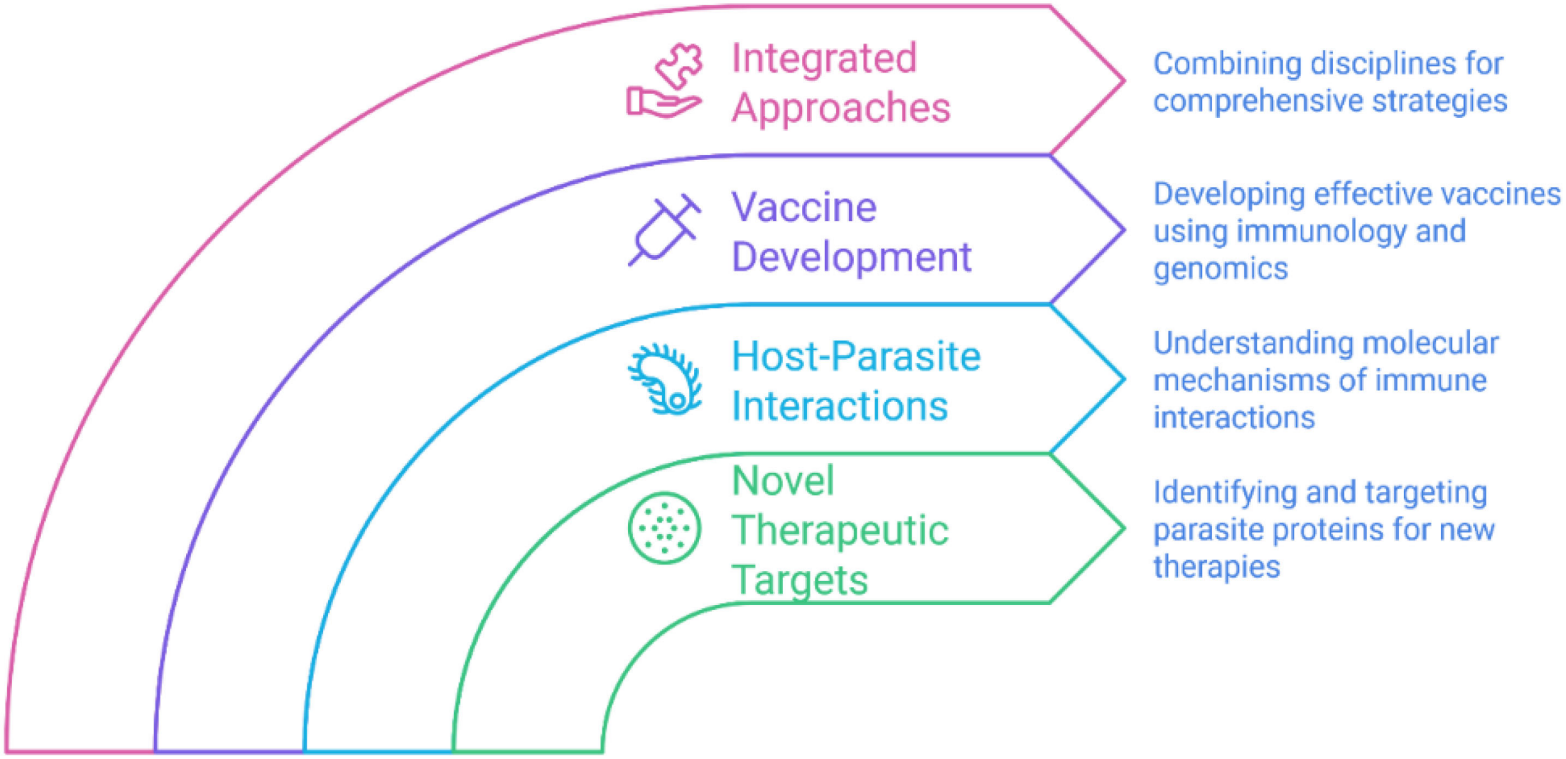
Advancements in immunology for food-borne parasites.

The topic of immunology related to food-borne parasites is quickly expanding, with several interesting avenues for future research (**Figure 1**):

1. Novel Therapeutic Targets: Finding and targeting the proteins involved in parasites’ immune escape might, in turn, develop new therapeutic medicines. For example, peptides from helminth CRT could be studied as possible vaccine targets or therapeutic agents.
2. Host-Parasite Interactions: Additional studies are necessary to illuminate the molecular bases of the interplay between food-borne parasites and the host’s immune system. This includes parasites that control host immune responses and identify the host characteristics linked to vulnerability or infection resistance.
3. Vaccine Development: Vaccines against food borne parasites are still a problem. Several new vaccine techniques — such as the exact targeting of parasite antigens or altering host immune responses — could be opened up by scientific progress in immunology and genetics.
4. Integrated Approaches: Immunology research could be combined with other fields such as genomics, proteomics and metabolomics to gain a more complete understanding of host-parasite interactions and to provide the basis for new prevention and treatment options.

In summary, recent advances in the laboratory of the immune system with regard to food borne parasites have greatly contributed to our understanding of these complex pathogen host interactions. The sector needs continuous research to develop effective measures to manage and prevent infectious diseases and to improve the world’s health in general.
